# Mechanical thrombectomy with intra-arterial alteplase provided better functional outcomes for AIS-LVO: a meta-analysis

**DOI:** 10.3389/fnins.2023.1137543

**Published:** 2023-07-06

**Authors:** Xingyu Yang, Zilan Wang, Huiru Chen, Youjia Qiu, Haiying Teng, Zhouqing Chen, Zhong Wang, Gang Chen

**Affiliations:** ^1^Department of Neurosurgery & Brain and Nerve Research Laboratory, The First Affiliated Hospital of Soochow University, Suzhou, Jiangsu Province, China; ^2^Department of Neurology, The First Affiliated Hospital of Soochow University, Suzhou, Jiangsu Province, China

**Keywords:** intra-arterial thrombolysis, alteplase, mechanical thrombectomy, acute ischemic stroke, functional outcomes after acute stroke

## Abstract

**Background:**

Several clinical trials have shown that intra-arterial thrombolysis using alteplase during mechanical thrombectomy (MT) has a better outcome than MT alone in ischemic stroke management. We performed the current meta-analysis to estimate the efficacy and safety of MT with intra-arterial alteplase therapy.

**Methods:**

The MEDLINE, Embase, Cochrane Library, and ClinicalTrials.gov databases were searched up to Mar. 2022 to identify the clinical trials that compared MT alone versus MT with intra-arterial alteplase therapy. STATA 16.0 was used for statistical analysis. The odds ratios (ORs) and 95% confidence intervals (95%CIs) were calculated with a random effect model.

**Results:**

Seven studies involving 1,083 participants were included. The primary outcomes were better functional outcomes, defined as a modified Rankin Scale (mRS) score between 0 and 2 at 90  days, and successful recanalization, defined as a modified thrombolysis in cerebral infarction (mTICI) score  ≥  2b. Compared to MT alone, MT with intra-arterial alteplase did not lead to higher mTICI scores (OR 1.58, 95%CI 0.94 to 2.67, *p* = 0.085, I^2^ = 16.8%) but did lead to better mRS (OR 1.37, 95%CI 1.01 to 1.86, *p* = 0.044). There was no increase in mortality or bleeding events in the overall or subgroup analyses.

**Conclusion:**

MT with intra-arterial alteplase did not improve the recanalization rate but provided better functional outcomes. The intervention did not increase adverse effects in any subgroup at the same time.

**Clinical trial registration:**

http://inplasy.com, identifier INPLASY202240027.

## Introduction

Acute ischemic stroke (AIS) is the leading cause of mortality and disability worldwide. AIS caused by large vessel obstruction (AIS-LVO) has a worse prognosis. Current evidence-based treatment options include intravenous thrombolysis (IVT) and mechanical thrombectomy (MT); however, these approaches still have several limitations (Ospel et al., 2020).

Intravenous alteplase has been showen substantially improved the outcomes of AIS patients and had become the first-line therapy for patients having AIS (Hacke et al., 2008). But the narrow time window of 4.5 h and several contradictions limited the recanalization efficacy of IVT for AIS-LVO (Bhatia et al., 2010).

MT has become the standard care for AIS with definitive efficacy and good safety (Powers et al., 2019). Intra-arterial thrombolysis (IAT) emerged initially in the PROACT trials (Prolyse in Acute Cerebral Thromboembolism) (del Zoppo et al., 1998). The Multicenter Randomized Clinical trial of Endovascular treatment for Acute ischemic stroke in the Netherlands (MR CLEAN) proved that intra-arterial therapy is effective and safe for AIS that is caused by proximal intracranial occlusion of the anterior circulation within 6 h after stroke onset (Berkhemer et al., 2015). The Interventional Management of Stroke (IMS) trials yielded promising outcomes in phase I and II studies but the phase III randomized clinical trial (RCT) came out with negative results (Investigators IS, 2004; Investigators IIT, 2007; Broderick et al., 2013). However, the role of IAT evolved from a primary therapy to adjunct or rescue therapy to mechanical thrombolysis.

It is necessary to evaluate the efficacy and safety of IVT, MT, IAT, and the combination of these therapies. A previous network meta-analysis comparing MT alone, IAT alone, MT + IVT, and IAT + IVT concluded that MT + IVT seemed to be the most effective strategy without increasing adverse effects (Hui et al., 2020). The efficacy of MT + IAT remained unclear. Another meta-analysis based on observational studies evaluating all modalities of MT and all categories of thrombolytics supported the potential role of IAT as an adjunct to MT (Chen et al., 2021).

Thrombolytic pharmaceuticals include urokinase, recombinant tissue plasminogen activator (rtPA, also named alteplase), and glycoprotein IIb/IIIa inhibitors, among which alteplase is the most well studied. Alteplase was first introduced in IVT and showed substantial improvement in outcomes.

Previous data comparing MT with intra-arterial alteplase and MT alone were mainly derived from observational studies. The two most commonly used efficacy outcomes were functional outcomes assessed by modified Rankin Scale (mRS) and recanalization assessed by modified Thrombolysis In Cerebral Infarction (mTICI) scale. The mRS scores were similar between the two groups or better in MT + IA tPA group. Recanalization showed heterogeneity in different studies. Heiferman et al. (2017) reported that MT with IA-tPA had a lower rate of mTICI = 2b but a higher rate of mTICI = 3. Anadani et al. (2019) also found a higher complete recanalization rate, while other studies showed no significant difference between the two groups. Consistently, these studies did not find an increase in adverse effects. Recently, a randomized controlled trial published the results of intra-arterial alteplase following successful MT (Renú et al., 2022). It concluded that intra-arterial alteplase as an adjunct therapy to MT resulted in a greater likelihood of excellent neurological outcomes at 90 days. We performed the current meta-analysis to estimate the efficacy and safety of MT with intra-arterial alteplase. We performed further subgroup analysis to investigate the potential value in specific patients.

## Methods

Before the project started, we designed the protocol following the PRISMA guidelines (Page et al., 2021). We have submitted our study protocol to the INPLASY register (No. INPLASY202240027).

### Eligibility and exclusion criteria

Eligibility Criteria: (i) participants: patients with AIS-LVO, (ii) intervention: MT with intra-arterial alteplase, (iii) Control: MT alone or MT with placebo, and (iv) outcomes: efficacy outcomes including the mRS and mTICI; safety outcomes including hemorrhage transformation and mortality. Included studies were not requested to have all the outcome data.

Exclusion Criteria: (i) study type: case reports or case series and (ii) active control (i.e., that is known to be an effective treatment as opposed to a placebo).

### Search strategy and information sources

Two independent investigators (XYY and ZLW) systematically searched the MEDLINE, EMBASE, Cochrane Library, and ClinicalTrial.gov databases, up to Mar. 2022 to identify relevant studies. “Alteplase”, “recombinant tissue plasminogen activator”, “mechanical thrombectomy” and “stroke” were used as search keywords. The detailed search strategies are presented in the Supplementary Table S1.

### Study selection and data collection

Two reviewers independently screened and evaluated all study records from the database search according to the eligibility criteria listed above. A third reviewer who did not participate in the process of data collection was consulted to resolve disagreements. The two reviewers extracted the following data using a standardized form: baseline information, inclusion and exclusion criteria, efficacy and safety outcome results, and conclusions.

### Risk of bias

Two reviewers assessed the risk of bias using the methodological index for nonrandomized studies (MINORS) tool. Disagreements between the two reviewers were resolved by consulting a third reviewer, Each study was checked with the 12-item MINORS scale to obtain a total score that represents the quality of the study. The RCT was assessed with Cochrane Collaboration risk of bias tool, which included the following domain: selection bias, performance bias, detection bias, attrition bias, reporting bias, and other potential biases. Each domain was classified as “low,” “high” or “unclear.”

### Statistical analysis

We used STATA 16.0 for data analysis. Statistical heterogeneity was estimated via the I^2^ statistic. All analyses used a random effect model. Heterogeneity was classified as low heterogeneity (I^2^ < 30%),moderate heterogeneity (30% <I^2^ <50%),substantial heterogeneity (I^2^ of 50% or more). Odds ratios (ORs) and 95% confidence interval (95% CI) were used for dichotomous variables and were presented with a Forest plot. All statistical tests were 2-tailed and significance was set at *p* < 0.05. Sensitivity analysis was used to explore the stability of the pooled results.

### Outcome of interest

Efficacy outcomes included functional outcomes assessed at 3 months by the and modified Rankin Scale (mRS) and recanalization assessed by the modified Thrombolysis In Cerebral Infarction (mTICI) scale. The good functional outcome was set as mRS 0 to 2. The successful recanalization was defined as mTICI≥2b.

Safety outcomes were assessed by determining the rate of adverse effects, including mortality, symptomatic intracerebral hemorrhage (sICH), parenchymal hemorrhage type 2 (PH-2), and any hemorrhage.

### Subgroup analysis

We performed subgroup analysis according to the baseline characteristics such as age, NIHSS score, and the timing of intra-arterial alteplase administration. We set two subgroup marks: (i) age above or below 70 years old and (ii) IAT as adjunct or rescue therapy to MT.

## Results

### Baseline characteristics

**Figure 1:**
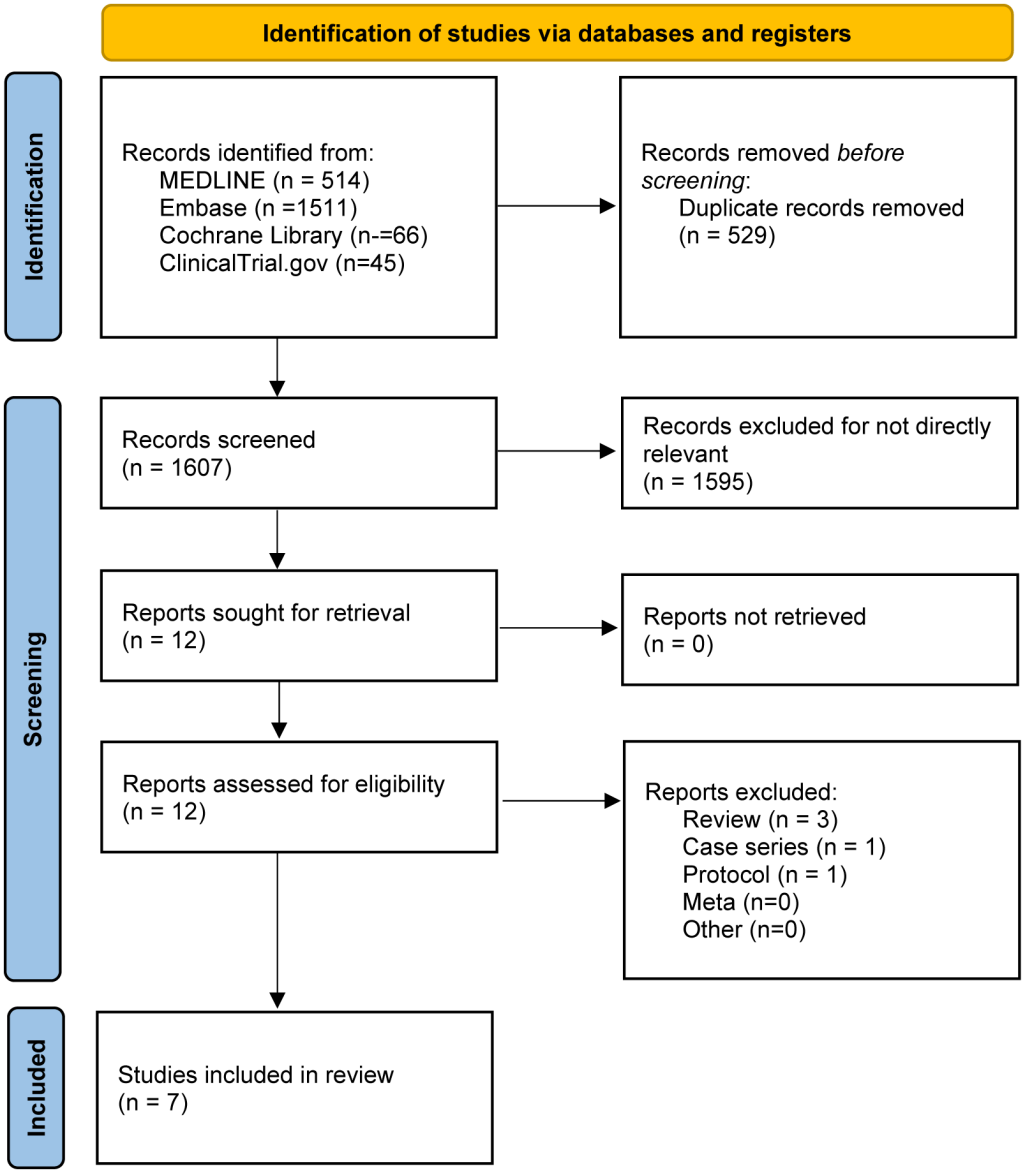
The study search, selection, and inclusion process.

We identified 2,136 references from the database searches. A total of 529 duplicates were removed. Irrelevant records were excluded after screening. Eligible articles were further assessed and seven studies (Lin et al., 2009; Heiferman et al., 2017; Yi et al., 2018; Anadani et al., 2019; Zaidi et al., 2019, 2021; Renú et al., 2022) were included in our final analysis (Figure 1). A total of 1,083 participants were pooled. The characteristics of each included study are listed in Table 1.

**Table 1:** Baseline characteristics of included studies.

Trials	Study type	Country/Center	Period	Timing of IAT to MT	Group	No. of participants	Age [Mean (SD)/Median (IQR)， years]	Sex [Male, *n* (%)]	NIHSS [Mean (SD)/Median (IQR)， years]
Lin et al. (2009)	Retrospective	USA	1998 to 2008	Before	MT + IAT	40	68 (13)	19 (47.5%)	17 (6–25)
					MT alone	18	71 (13)	10 (55.6%)	18 (10–29)
Heiferman et al. (2017)	Prospective	USA	Jan 2015 to Mar 2016	Adjunct	MT + IAT	28	67 (56–74)	10 (36%)	20 (15–25)
					MT alone	12	69 (63–78)	5 (42%)	18 (14–22)
Anadani et al. (2019)	Retrospective	USA	Nov 2014 to Jan 2018	Rescue	MT + IAT	419	66.1 (15)	34 (50.7%)	15.2 (7.6)
					MT alone	67	68.2 (14.2)	205 (48.9%)	15.9 (7.4)
Yi et al. (2018)	Retrospective	China	2015 to 2017	Adjunct	MT + IAT	37	66 (13)	17 (45.9%)	18 (11–23)
					MT alone	56	65 (11)	30 (53.6%)	18 (10–28)
Zaidi et al. (2019)	Retrospective	24 sites in the USA	Mar 2012 to Feb 2013	Rescue	MT + IAT	37	70.7 (15.4)	21 (56.8%)	17.5 (14–22)
					MT alone	44	69.1 (17.8)	22 (50%)	19 (13–21)
Zaidi et al. (2021)	Prospective	55 centers in the USA	Aug 2014 to Jun 2016	Rescue	MT + IAT	129	68 (15.2)	69 (53.5%)	17.0 ± 5.5
					MT alone	83	65.9 (15.3)	53 (63.9%)	17.6 ± 5.7
Renú et al. (2022)	RCT	7 centers in Spain	Dec 2018 to May 2021	Adjunct	MT + IAT	61	73 (71–76)	33 (54%)	14 (8–20)
					MT + placebo	52	73 (69–77)	28 (54%)	14 (10–20)

### Efficacy outcomes

**Figure 2:**
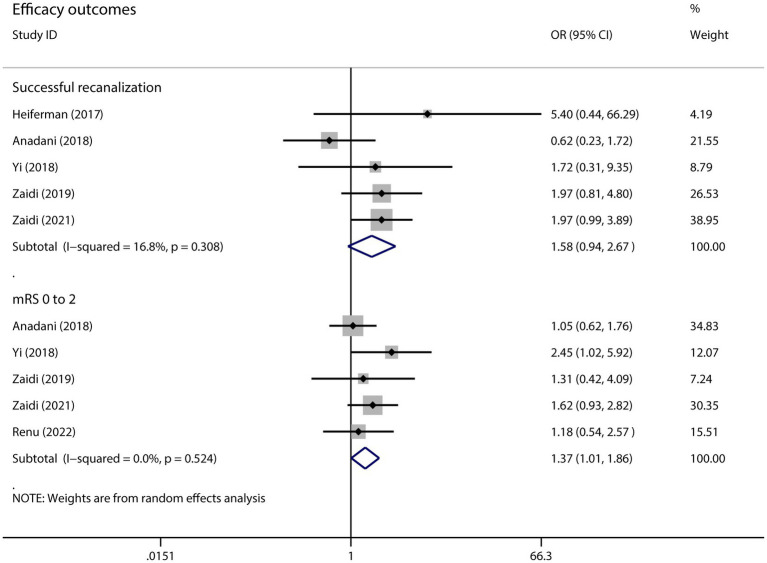
The pooled OR and 95%CI of efficacy outcomes. The diamond indicates the estimated OR and 95%CI and the square indicated the weight of each study.

We combined data for the outcome of recanalization using OR with random effects model. Compared to MT alone, MT with intra-arterial alteplase did not show higher recanalization rate (OR 1.58, 95%CI 0.94–2.67, *p* = 0.085, I^2^ = 16.8%) but yielded better functional outcome of mRS 0 to 2 (OR 1.37 95%CI 1.01–1.86, *p* = 0.044, I^2^ = 0.0%) (Figure 2).

Subgroup analysis did not yield any positive result. The Forrest plot of each subgroup was presented in Supplementary Figure S1.

### Safety outcomes

**Figure 3:**
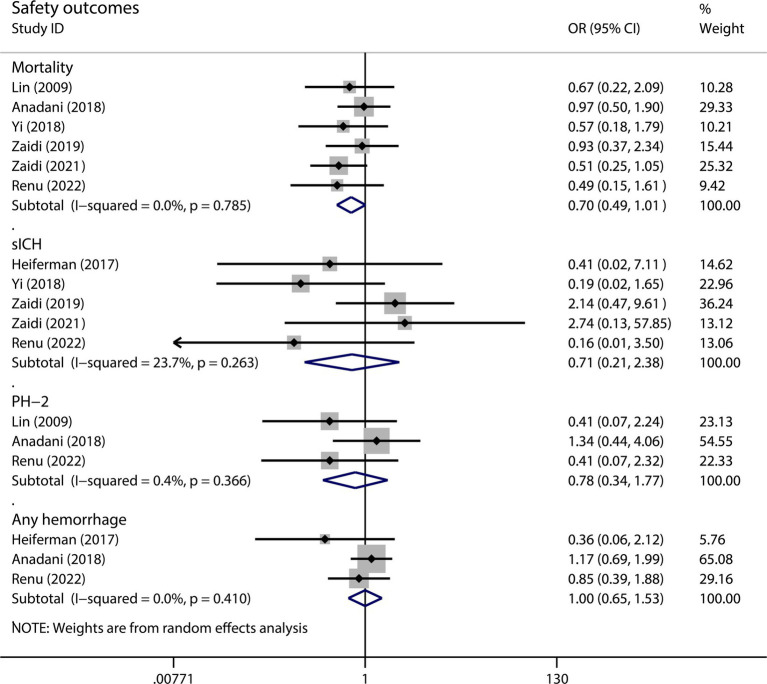
The pooled OR and 95%CI of safety outcomes. The diamond indicates the estimated OR and 95%CI and the square indicated the weight of each study.

Among the four analyzed indicators of adverse effects, we did not observe significant differences between the two groups (Figure 3). The administration of intra-arterial alteplase during MT did not increase the risk of mortality or hemorrhage. The results were as follows: mortality rate (OR 0.70, 95%CI 0.49–1.01, *p* = 0.055, I^2^ = 0.0%), sICH (OR 0.71, 95%CI 0.21–2.38, *p* = 0.584, I^2^ = 23.7%), PH-2 (OR 0.78, 95%CI 0.34–1.17, *p* = 0.550, I^2^ = 0.4%), and any hemorrhage (OR 1.00, 95%CI 0.65–1.53, *p* = 0.998, I^2^ = 0.0%). Subgroup analysis also revealed negative results (Table 2). The analysis of sICH patients aged>70 years old showed a higher level of heterogeneity (I^2^ = 55.3%). We performed a sensitivity analysis and the results are shown in Supplementary Figure S2.

**Table 2:** The results of subgroup analysis.

	Efficacy outcomes				Safety outcomes			
	Good functional outcome		Recanalization		Mortality		sICH	
	OR (95%CI)	*p* value	OR (95%CI)	*p* value	OR (95%CI)	*p* value	OR (95%CI)	*p* value
Age								
Age>70	1.221 (0.642, 2.320)	0.543	NA	NA	0.728 (0.351, 1.511)	0.394	0.840 (0.072, 9.768)	0.889
Age<70	1.466 (0.942, 2.282)	0.090	1.457 (0.693, 3.063)	0.322	0.692 (0.455, 1.053)	0.085	0.448 (0.101, 1.996)	0.292
IAT as adjunct or rescue therapy to MT								
Adjunct	1.650 (0.808, 3.368)	0.169	2.458 (0.603, 10.014)	0.209	0.532 (0.234, 1.209)	0.132	0.229 (0.051, 1.019)	0.053
Rescue	1.284 (0.896, 1.840)	0.173	1.436 (0.725, 2.844)	0.299	0.762 (0.493, 1.176)	0.219	2.242 (0.582, 8.639)	0.241

### Risk of bias

We included three retrospective studies, three prospective studies, and one RCT in this meta-analysis. The risk of bias in observational studies is listed in Table 3. The total points ranged from 17 to 20. The RCT (Renú et al., 2022) was categorized as “low risk of bias” for each domain.

**Table 3:** A summary table for risk of bias item assessed by MINORS scale of each study.

Item	Lin et al. (2009)	Heiferman et al. (2017)	Anadani et al. (2019)	Yi et al. (2018)	Zaidi et al. (2019)	Zaidi et al. (2021)
1. A clearly stated aim	2	2	2	2	2	2
2. Inclusion of consecutive patients	2	2	2	2	2	2
3. Prospective collection of data	0	2	2	0	0	2
4. Endpoints appropriate to the aim of the study	2	2	2	2	2	2
5. Unbiased assessment of the study endpoint	1	0	0	0	0	0
6. Follow-up period appropriate to the aim of the study	2	2	2	2	2	2
7. Loss to follow up less than 5%	2	2	2	2	1	1
8. Prospective calculation of the study size	0	0	0	0	0	0
9. An adequate control group	2	2	2	2	2	2
10. Contemporary groups	2	2	2	2	2	2
11. Baseline equivalence of groups	2	2	2	2	2	2
12. Adequate statistical analysis	2	2	2	2	2	2
13. Total points	19	20	20	18	17	19

The items are scored 0 (not reported), 1 (reported but inadequate), or 2 (reported and adequate). The global ideal scores are 16 for non-comparative studies and 24 for comparative studies.

## Discussion

We pooled 1,083 participants from seven studies in our meta-analysis to estimate the safety and efficacy of intra-arterial alteplase during MT. The results suggested that compared to MT alone, MT with intra-arterial alteplase led to better functional outcomes but did not improve recanalization. Further more, there was no increase in adverse effects. Overall, intra-arterial alteplase showed good efficacy and safety outcomes.

The efficacy outcomes were assessed by widely used tools, the mRS was used to assess functional outcome and the mTICI was used to assess recanalization. The MT with intra-arterial alteplase group did not show a higher successful recanalization rate but did show a higher rate of good functional outcomes. This phenomenon might be explained by the limitations of the mTICI scale. The reperfusion rate is typically calculated by the operator at the end of the procedure, which could be influenced by experience. Patients might benefit from intra-arterial thrombolysis owing to the better reperfusion that is not reflected in mTICI score. The most recent TICI (expanded TICI, eTICI) was published in 2019. The expanded TICI scale divided reperfusion extent into 7 grades. It provided cut-off points by demonstrating excellent reliability for distinguishing eTICI 2b50 and 2b67. The efficacy of eTICI was examined using a large multinational dataset (Liebeskind et al., 2019). Renú et al. (2022) utilized the recent iteration to obtain a more objective reperfusion assessment.

The most important safety concern about the addition of intra-arterial alteplase to MT might be the potential hemorrhagic transformation, especially intracerebral hemorrhage. The hemorrhagic risk was estimated by the rate of hemorrhage events such as sICH, PH-2, and any hemorrhage in most studies. Our results did not show any significant difference in these hemorrhagic indices, nor did the subgroup analysis. The results are somewhat unexpected but not unreasonable. Thrombolytic agents truly have a direct effect on hemorrhage tendency, but the mechanisms of ICH secondary to intra-arterial revascularization therapies in AIS are complex. The blood–brain barrier damage triggered by ischemia is another pivotal process (Mokin et al., 2012). The risk of hemorrhage and the benefit of timely reperfusion need equilibrium. Another meta-analysis comparing MT with adjunctive intra-arterial thrombolysis also obtained similar results that sICH rates were not increased (Diprose et al., 2021). However, the included studies used different classification systems of sICH that might cause heterogeneity and bias. The results also showed no difference in mortality between the two groups. MT with IAT tended to decrease mortality compared to MT alone.

Our study is the first meta-analysis that focused on the combination of specific intra-arterial thrombolytic alteplase and MT by incorporating the data from the most recent RCT. The results and conclusions are consistent with the outcomes of the RCT and most previous studies. The level of heterogeneity was low in the majority of outcomes analyzed herein. The subgroup analysis of sICH in age>70 showed substantial heterogeneity. But only two studies, Zaidi et al. (2019) and Renú et al. (2022), were included in this subgroup. Zaidi et al. (2019) was a retrospective study while Renú et al. (2022) was the most updated RCT, which might cause heterogeneity.

The CHOICE trial (Chemical Optimization of Cerebral Embolectomy) was the first RCT to publish the outcomes of intra-arterial alteplase following successful MT. The RCT differs from the previous observational studies because it used successful recanalization as an indicator of eligibility for intra-arterial alteplase administration. It also used the most updated expanded Thrombolysis In Cerebral Infarction (eTICI) index to assess recanalization. Therefore, the RCT could not be included in the analysis of recanalization herein. Another regretful thing is that CHOICE trial was terminated early and did not reach its expected recruitment target due to the COVID-19 pandemic. The most updated RCT had a relatively small sample size and thus weighted less heavily in this meta-analysis.

Previous RCTs about intra-arterial therapy, such as PROACT and MR CLEAN, are not included in our meta-analysis due to different study designs. A more recent observational study of MR CLEAN Registry analyzed the participants receiving IA thrombolytics following EVT and showed neutral results (Collette et al., 2023). This study got similar rate of favorable outcome (defined as mRS 0–2) between the groups with or withour IA thrombolytics and found less reperfusion rate in patients treated with IA thrombolytics, which are inverse to our results. But the neutral results about sICH are consistent with us.

There are some other limitations in our meta-analysis. A certain percentage of participants received IVT, the impact of which could not be ignored, but we could not perform a subgroup analysis of these subsets due to the lack of data. The occlusion location of vessels and the door-to-needle time or onset-to-recanalization time might also influence the outcomes, but fewer studies have reported detailed data on the specific patients’ prognoses.

IAT had potential efficacy as adjunctive therapy to MT and could not be discarded. The evaluation of other currently used thrombolytics such as urokinase and tirofiban is also necessary. But present data about intra-arterial thrombolysis for AIS management were still mainly derived from observational studies. More RCTs are needed to obtain higher-quality evidence for the administration of intra-arterial thrombolysis. We are looking forward to the results from ongoing research, for example, the TECNO trial (NCT05499832) aiming at assessing safety and efficacy of intra-arterial Tenecteplase for noncomplete reperfusion of intracranial occlusions.

## Conclusion

Compared to MT alone, MT with intra-arterial alteplase did not improve the recanalization rate but provided better functional outcomes. The intervention did not increase hemorrhage or mortality risk. Thus, MT with intra-arterial alteplase could be a potential therapy for AIS-LVO.

## Data availability statement

The original contributions presented in the study are included in the article/Supplementary material, further inquiries can be directed to the corresponding authors.

## Author contributions

ZhW was the principal investigator. ZC designed the study protocol. XY, ZiW, and HC searched the databases, screened the studies, and analyzed the data. YQ, HT, and GC revised the manuscript and polished the language. All authors contributed to the article and approved the submitted version.

## Funding

This work was supported by the National Natural Science Foundation of China (Granted to Zhong Wang, Grant No. 81873741) and the Natural Science Foundation of Jiangsu Province (Granted to Zhouqing Chen, Grant No. BK 20200203), Suzhou Science and Technology Development Plan Projects (Granted to ZW, Grant No. SS202057).

## Conflict of interest

The authors declare that the research was conducted in the absence of any commercial or financial relationships that could be construed as a potential conflict of interest.

## Publisher’s note

All claims expressed in this article are solely those of the authors and do not necessarily represent those of their affiliated organizations, or those of the publisher, the editors and the reviewers. Any product that may be evaluated in this article, or claim that may be made by its manufacturer, is not guaranteed or endorsed by the publisher.

## Glossary

AIS: Acute ischemic stroke
CHOICE: Chemical Optimization of Cerebral Embolectomy
IAT: Intra-arterial thrombolysis
IMS: Interventional Management of Stroke
IVT: Intravenous thrombolysis
LVO: Large vessel occlusion
MINORS: Methodological index for non-randomized studies
MR CLEAN: Multicenter Randomized Clinical trial of Endovascular treatment for Acute ischemic stroke in the Netherlands
mRS: modified Rankin Scale
mTICI: modified Thrombolysis In Cerebral Infarction
MT: Mechanical thrombectomy
NIHSS: National Institute of Health stroke scale
PH-2: Parenchymal hemorrhage type 2
PROACT: Pyrolyse in Acute Cerebral Thromboembolism
RCT: Randomized clinical trial
rtPA: Recombinant tissue plasminogen activator
sICH: Symptomatic intracerebral hemorrhage
