# Supplementary material for: Mechanical thrombectomy with intra-arterial alteplase provided better functional outcomes for AIS-LVO: a meta-analysis
Source: Front Neurosci. 2023 Jul 6;17:1137543. doi: 10.3389/fnins.2023.1137543 (PMC10403059; doi:10.3389/fnins.2023.1137543)

Mechanical thrombectomy with intra-arterial alteplase provided better functional outcome for AIS-LVO: a meta-analysis from clinical trials

Supplemental Materials

Table S1 Search strategy and results

Database	Records	Search strategy
MEDLINE	514	(((((alteplase[Title/Abstract]) OR (recombinant tissue plasminogen activator[Title/Abstract])) OR (rtPA[Title/Abstract])) OR (tPA[Title/Abstract])) AND (mechanical thrombectomy[Title/Abstract])) AND (stroke[Title/Abstract])
Embase	1511	('alteplase'/exp OR alteplase OR 'recombinant tissue plasminogen activator':ab,ti OR rtpa:ab,ti OR tpa:ab,ti) AND 'mechanical thrombectomy':ab,ti AND stroke:ab,ti
Cochrane Library	66	alteplase in Title Abstract Keyword AND mechanical thrombectomy in Title Abstract Keyword AND stroke in Title Abstract Keyword - (Word variations have been searched) (alteplase):ti,ab,kw AND (mechanical thrombectomy):ti,ab,kw AND (stroke):ti,ab,kw
ClinicalTrials.gov	45	alteplase and mechanical thrombectomy | Stroke

Table S2 Inclusion and exclusion criteria, conclusion, data acquisition time and outcome assessments of the included studies

**Trials**	**Lin2009**
Inclusion Criteria	Consecutive cases of acute ischemic stroke attributable to TICA occlusions treated endovascularly
Exclusion Criteria	Unreported
Efficacy Outcomes	Recanalization assessed by TIMI.
Safety Outcomes	1.Parenchymal hemorrhage type 1 and type 2.<br>2.Mortality.
Conclusion	Combined mechanical and intra-arterial pharmacological therapy is associated with higher recanalization rates than either intervention alone in acute internal carotid artery terminus occlusion revascularization.
Data acquisition time	The interventionalists determined the recanalization status at the end of the case.

Trials	Heiferman2017
Inclusion Criteria	Patients who underwent MT for large-vessel occlusion by senior authours.
Exclusion Criteria	Unreported
Efficacy Outcomes	Revascularization assessed by mTICI.
Safety Outcomes	Hemorrhage conversion.
Conclusion	Concomitant IA tPA thrombolysis is a safe adjunct to MT treatment of acute LVO with and without concomitant use of IV tPA. Further investigation into the efficacy of this method and optimal use is warranted. With a renewed fervor for interventional treatment of stroke, we must clarify those methods that will lead to best outcomes for this heterogeneous patient population.
Data acquisition time	Revascularization was assessed within 24 hours of the procedure by CT and MRI.<br>Symptomatic hemorrhages were noted when patients had a decline in neurologic examination.

Trials	Anadani2018
Inclusion Criteria	Patients in the prospectively maintained thrombectomy dataset who received MT at a large tertiary-care center.
Exclusion Criteria	Unreported.
Efficacy Outcomes	The functional outcome assessed by mRS and the recanalization rate assessed by mTICI.
Safety Outcomes	The rate of hemorrhagic complications, including hemorrhagic transformation and subarachnoid hemorrhage.
Conclusion	IA-tPA administration during MT was not associated with increased risk of hemorrhage in selected patients with incomplete recanalization after thrombectomy.
Data acquisition time	Functional outcomes were assessed at 90 days.<br>Hemorrhagic complications were determined within 72 hours of procedure.

**Trials**	**Yi2018**
Inclusion Criteria	1.acute anterior circulation ischemic stroke due to intracranial large artery embolism occlusion<br>2.Treatment using stent retrieval<br>3.onset-to-presentation time within 8 hours<br>4.age greater than 18 years<br>5.pre-stroke mRS score of 0-1
Exclusion Criteria	The cause of stroke was dissection, Moyamoya disease, vasculitis, tandem occlusion, or intracranial arteriosclerosis.
Efficacy Outcomes	Neurologic function assessed by mRS at 90 days.<br>Brain tissue reperfusion assessed by mTICI.
Safety Outcomes	Symptomatic intracranial hemorrhage (sICH), distal embolism, artery re-occlusion, neurologic disability at 90 days, and death.
Conclusion	Relative to the use of a stent retriever alone, the use of a stent retriever combined with intra-arterial rt-PA at the initial pass for mechanical thrombectomy shortens the procedure time, decreases the number of passe of the stent, lowers the likelihood of an embolic event, improves the rate of recanalization with 1 or 2 passes, improves patient prognosis and does not increase the adverse event rate.
Data acquisition time	Functional outcomes were assessed at 90 days.<br>Brain tissue reperfusion was assessed immediately after the operation.<br>Symptomatic intracranial hemorrhage was defined within 36 hours after treatment.

**Trials**	**Zaidi2019 (NASA Registry, NCT02239640)**
Inclusion Criteria	Unreported
Exclusion Criteria	Unreported
Efficacy Outcomes	The functional outcome assessed by mRS and the recanalization rate assessed by mTICI.
Safety Outcomes	Symptomatic ICH and mortality.
Conclusion	the use of intraarterial rtPA as RT yielded a similar safety profile, with comparable rates of good functional outcome at 90 days. We recommend that future studies, including large prospective randomized clinical trials, are needed to establish the proper patient population and protocol for intraarterial rtPA administration and to determine if intraarterial rtPA is more advantageous as an adjunctive or RT.
Data acquisition time	Recanalization on the angiogram at the end of the procedure.<br>Mortality and mRS at 90 days.<br>Symptomatic ICH on the 24h follow-up head CT.

**Trials**	**Zaidi2021 (STRATIS registry, NCT02239640)**
Inclusion Criteria	1. confirmed, symptomatic intracranial large-vessel occlusion with associated symptoms;<br>2. an NIHSS score of 8 to thirty;<br>3. use of the Medtronic market-released neurothrombectomy device as the initial device;<br>4. premorbid mRS of #1;<br>5. treatment within 8 hours of stroke onset.
Exclusion Criteria	Unreported
Efficacy Outcomes	The functional outcome assessed by mRS, recanalization assessed by mTICI and the mean time of onset to groin puncture.
Safety Outcomes	The incidence of symptomatic intracranial hemorrhage and mortality.
Conclusion	Use of intra-arterial rtPA as a rescue therapy after unsuccessful mechanical thrombectomy was not associated with an increased risk of symptomatic intracranial hemorrhage or mortality. Randomized clinical trials are needed to understand the safety and efficacy of intra-arterial thrombolysis as a rescue therapy after mechanical thrombectomy.
Data acquisition time	MRS at 90 days. Adverse effects up to 90 days post procedure.

Trials	Renu2022
Inclusion Criteria	1.Participants were aged 18 years or older;<br>2.had a large vessel occlusion in the anterior, middle, or posterior cerebral artery;<br>3. were treated with thrombectomy within 24 hours after the point when they were last seen well;<br>4. had a post thrombectomy eTICI score of 2b50 or greater as judged by local investigators;<br>5. had been able to carry out usual activities in their daily life without support before the stroke.
Exclusion Criteria	1.any contraindication to the use of intravenous alteplase per local and national guidelines (except time to therapy)<br>2.a National Institutes of Health Stroke Scale (NIHSS) score on admission of greater than 25, (range, 0-42, with higher values indicating more severe deficit).<br>3.Complete clinical recovery in the angiography suite during the procedure
Efficacy Outcomes	The proportion of patients with a score of 0 or 1 on the modified Rankin Scale at 90days.<br>The proportion of patients with improved angiographic findings; the infarct expansion ratio of final infarct to initial ischemic tissue volumes; the proportion of patients with an infarct expansion ratio greater than 1; and the infarction volume at 24 to 48 hours after stroke onset.<br>The tertiary efficacy outcomes included the proportion of patients with a Barthel Scale score of 95 to 100 at day 90; the proportion of ischemic worsening (≥4-point increase on the NIHSS score) within 48 hours to 72 hours of stroke onset; and quality of life as measured with the EuroQol 5-Dimension 3-Level Self-Report Questionnaire (EQ-5D-3L) at 90 days.
Safety Outcomes	The advers eevents measured included incidence of symptomatic intracranial hemorrhage, defined as neurological deterioration (≥4-point increase on the NIHSS score) within 24 hours after treatment and evidence of intracranial hemorrhage on imaging studies, and death at 90 days.
Conclusion	Among patients with large vessel occlusion acute ischemic stroke and successful reperfusion following thrombectomy, the use of adjunct intra-arterial alteplase compared with placebo resulted in a greater likelihood of excellent neurological outcome at 90 days. However, because of study limitations, these findings should be interpreted as preliminary and require replication.
Data acquisition time	Clinical assessments were performed at baseline, at 24 hours and 48 hours after randomization, at 5 to 7 days (or at discharge if earlier), and at 90 days.<br>Neurovascular images were assessed at admission, 24 hours and 48 hours. Angiograms were assessed pre-treatment and post-treatment.

Figure.S1 The pooled OR and 95%CI of subgroup analysis

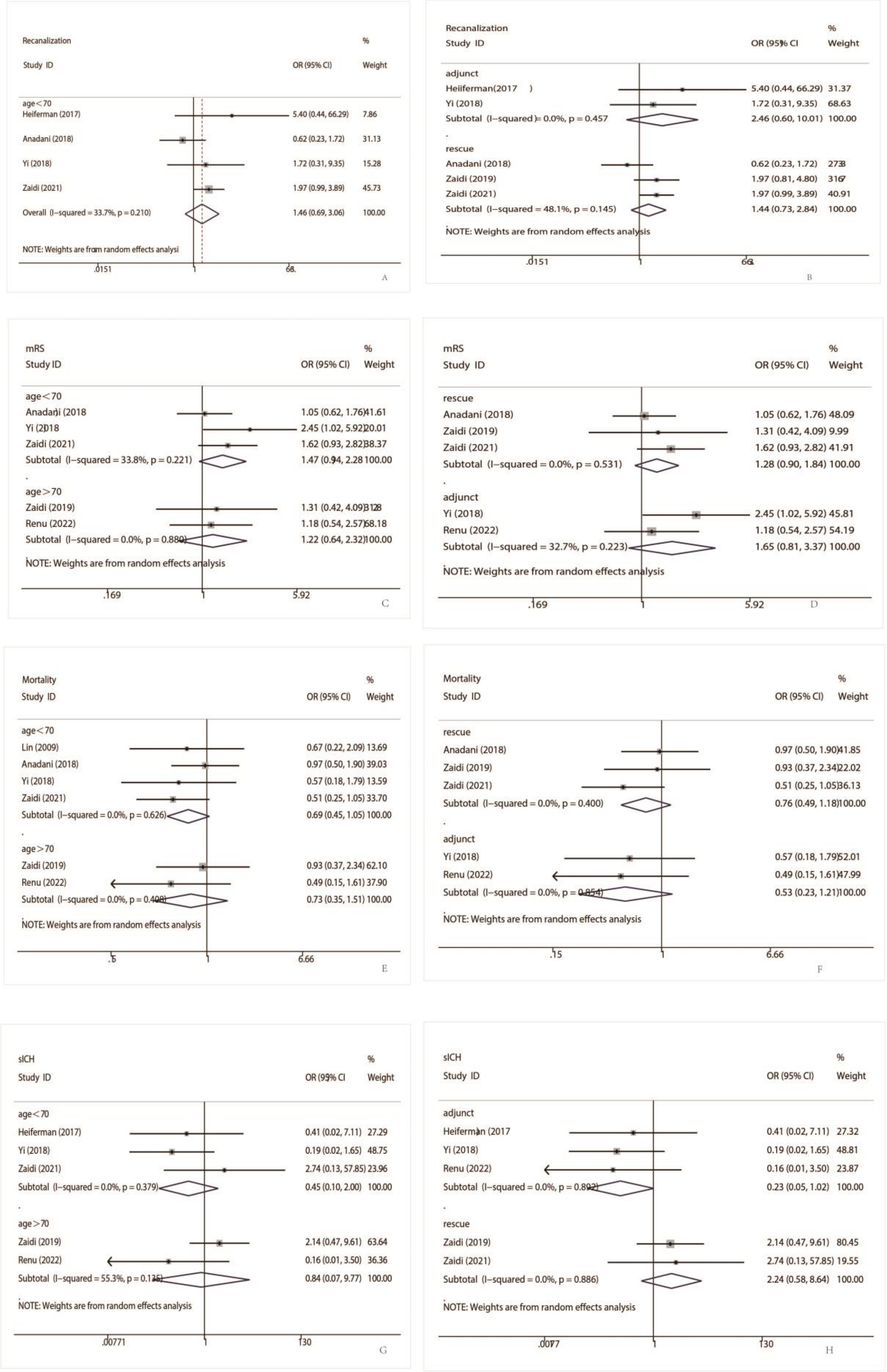

Subgroup analysis of recanalization, mRS, mortality and sICH, according to baseline age and timing of intra-arterial alteplase. A, recanalization of age; B, recanalization of timing; C, mRS of age; D, mRS of timing; E, mortality of age; F, mortality of timing; G, sICH of age; H, sICH of timing.

Fig.S2 Sensitivity analysis for the subgroup of sICH with age > 70

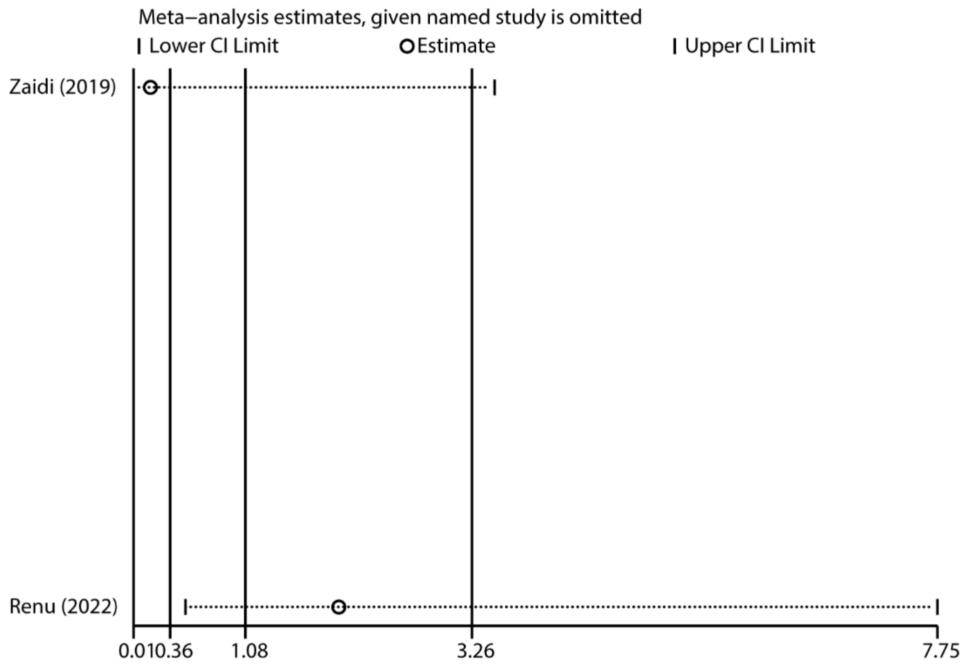

Supplement: Supplementary file 1 [file Data_Sheet_1.PDF]
